# Economics achieved through the use of artificial intelligence–powered contouring solutions in a network of oncology clinics in low- and middle-income countries

**DOI:** 10.3389/fonc.2026.1809864

**Published:** 2026-04-27

**Authors:** Vibhor Gupta, Maithili Sharma, Rahul Lal Chowdhary, Sumeet Aggarwal, Mohal Lal, Midhun Murali, Manikumar Singamsetty, Suresh Chaudhari, Man Gobinda Chowdhary, Sushil Beriwal

**Affiliations:** 1 American Oncology Institute, Hyderabad, India; 2 American Oncology Institute, Jalandhar, India; 3 American Oncology Institute, Hisar, India; 4 American Oncology Institute, Calicut, India; 5 American Oncology Institute, Mangalagiri, India; 6 Allegheny Health Network, Pittsburgh, PA, United States; 7 Allegheny Health Network and Varian Medical Systems, Palo Alto, CA, United States

**Keywords:** artificial intelligence, autocontouring, cost analysis, low- and middle-income countries, radiation oncology, workflow efficiency

## Abstract

**Background:**

Contouring of target volumes and organs-at-risk (OARs) is among the most time-intensive steps in radiation therapy planning. Artificial intelligence (AI)–based autocontouring has the potential to improve efficiency and reduce clinician workload, particularly in high-volume settings.

**Methods:**

This multi-center study evaluated time and labor cost savings following implementation of an AI-powered autocontouring solution (AI-Rad Companion Organs RT, Siemens Healthineers) across 18 oncology centers in a low- and middle-income country. Six physicians assessed 116 radiotherapy planning cases across multiple anatomical sites. Time required for manual contouring and post-autocontour editing was recorded. Time savings were converted into monetary value using three clinician cost scenarios, accounting for a notional software usage cost.

**Results:**

Mean time savings across all cases was 13.8 minutes. After accounting for software costs, net savings ranged from INR 37 to INR 670 per case depending on clinician cost assumptions.

**Conclusion:**

AI-based autocontouring significantly reduces clinician workload and delivers measurable labor cost savings in routine radiation oncology practice, supporting adoption in LMIC settings.

## Introduction

Radiation therapy (RT) is a cornerstone of cancer management and requires meticulous treatment planning to maximize tumor control while minimizing dose to surrounding healthy tissues. Following CT simulation, clinicians delineate target volumes and organs-at-risk (OARs), a process that is both clinically critical and highly time-consuming.

The increasing adoption of advanced techniques such as intensity-modulated radiation therapy (IMRT), volumetric modulated arc therapy (VMAT), and stereotactic body radiation therapy (SBRT) has further increased the number and complexity of structures requiring contouring (1). This growing burden represents a significant operational cost in terms of highly skilled clinician time.

AI-based autocontouring systems employ deep learning algorithms trained on large datasets of expertly annotated images to automatically generate contours. While improved consistency and standardization are often cited motivations for adoption, the associated reduction in clinician time represents a direct opportunity for measurable economic benefit. This study quantifies the time and labor cost savings associated with implementation of an AI-based autocontouring solution across a large oncology network in a low- and middle-income country.

## Materials and methods

**Figure 1:**
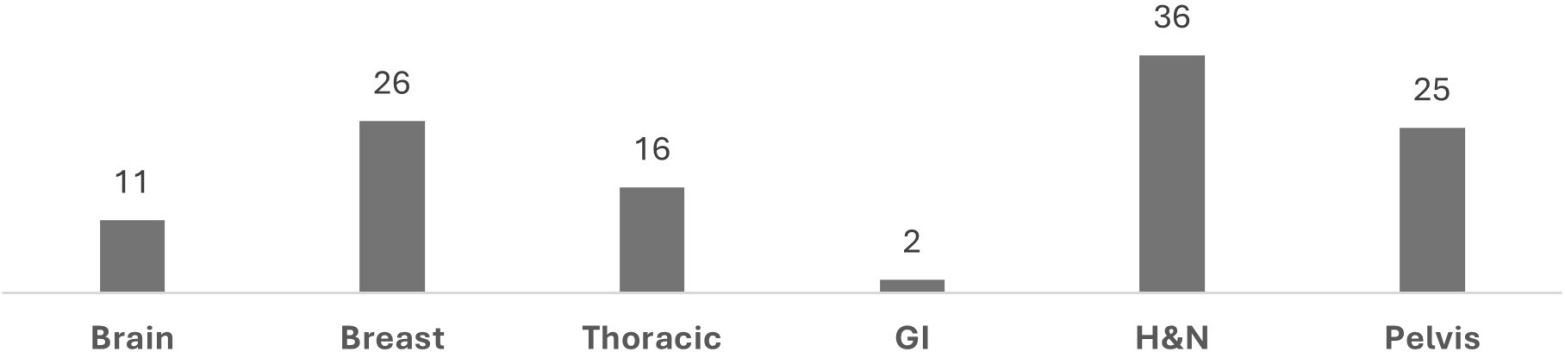
Number of cases by anatomical region.

A total of 116 patients undergoing radiotherapy planning were selected consecutively and included in the analysis: 11 brain, 36 head and neck, 26 breast, 16 thoracic, 2 gastrointestinal, and 25 pelvic cases (**Figure 1**). The Gastrointestinal cohort had limited statistical significance as there were only two cases. Six experienced physicians from multiple centers within the network participated in the study.

For each case, physicians recorded the time required for manual OAR contouring. Automated contours were then generated using the AI-Rad Companion Organs RT (AIRC) application. The same physicians reviewed the AI-generated contours based on published data including RTOG, ESTRO and others. They also recorded the time required for editing. Contour usability was scored on a four-point clinical scale ranging from complete usability without edits to complete re-contouring.

Time savings were calculated as the difference between manual contouring time and AI contour editing time. Monetary value of time saved was estimated using three clinician cost scenarios: low (INR 1,250/hour), optimal (INR 2,500/hour), and high (INR 4,000/hour). A notional AIRC usage cost of INR 250 per case was subtracted to calculate net savings. No additional costs were considered as there are no infrastructure, implementation, maintenance, or training costs involved. Statistical analysis was performed using SPSS version 2024. Paired T-test were used to compare manual vs AI-assisted contouring times.

## Results

The mean time saving across all cases was 13.8 minutes. Organ-specific time savings ranged from 5.5 minutes for gastrointestinal cases to over 16 minutes for breast and thoracic cases (**Table 1**). When clinician costs were applied, net per-case savings ranged from INR 37 for low-cost clinicians to INR 670 for high-cost clinicians, with an average saving of INR 325 per case under the optimal cost scenario (**Table 2**).

**Table 1:** Organ-wise summary of manual and automated contouring times.

Site	Manual mean (min)	Manual IQR (min)	Auto mean (min)	Auto IQR (min)	P-value
Brain	10.09	7.50–13.50	1.36	1.00–1.25	<0.01
Head & Neck	17.83	14.00–23.50	4.32	1.00–8.00	<0.01
Breast	18.71	9.00–24.00	2.07	0.38–3.00	<0.01
Thoracic	17.13	9.00–18.50	0.79	0.00–1.50	<0.01
GI	5.50	5.25–5.75	0.00	0.00–0.00	0.06
Pelvis	14.60	7.00–20.00	2.44	1.00–4.00	<0.01

**Table 2:** Organ-wise summary of cost savings with and without AIRC costs.

Site	Cases	Optimal (INR)	High (INR)	Low (INR)	Optimal net*	High net*
Brain	11	398	636	199	148	386
Breast	26	693	1,109	347	443	859
Thoracic	16	681	1,089	340	431	839
GI	2	229	367	115	(21)	117
Head & Neck	36	563	901	281	313	651
Pelvis	25	507	811	253	257	561
All Cases	116	575	920	287	325	670

* Considering notional cost of AIRC at INR 250/ case.

## Discussion

This multi-center analysis demonstrates that AI-powered autocontouring can deliver clinically meaningful reductions in contouring time and associated labor costs across a range of disease sites in routine radiation oncology practice (2–9). By focusing on real-world workflow metrics, this study provides pragmatic evidence of the operational value of AI-assisted contouring in high-volume LMIC settings.

The observed mean time saving of 13.8 minutes per case represents a substantial efficiency gain when applied at scale. In institutions managing thousands of radiotherapy plans annually, such gains translate into significant recovery of clinician time. The largest benefits were observed in breast and thoracic cases, likely reflecting more standardized anatomy and OAR definitions.

From an economic perspective, AI-assisted contouring remained favorable even after accounting for software usage costs. Although per-case savings appear modest, the cumulative impact in high-throughput environments is considerable. Importantly, in LMIC settings, reclaimed clinician time may improve access to care and mitigate workforce limitations.

These findings are consistent with prior reports evaluating commercial AI-based autocontouring systems, which have demonstrated substantial reductions in contouring time across multiple anatomical regions. Beyond direct cost savings, indirect benefits may include improved workflow consistency, enhanced quality assurance, and reduced clinician fatigue.

Limitations include reliance on subjective clinical usability scoring rather than geometric similarity metrics, assumed software costs, and limited generalizability to healthcare systems with different workforce models. Nevertheless, the pragmatic design and multi-center nature of this study strengthen its relevance for real-world implementation.

## Conclusion

AI-driven autocontouring offers a scalable and economically viable solution to improve efficiency in radiation oncology workflows. In high-volume LMIC settings performing approximately 10,000 planning cases annually, cumulative labor cost savings are substantial. Institutions should consider local workforce models and software costs when evaluating adoption.

## Data Availability

The raw data supporting the conclusions of this article will be made available by the authors, without undue reservation.
